# A novel and practical method to add video monitoring to tilt table testing

**DOI:** 10.1093/europace/euac193

**Published:** 2022-11-10

**Authors:** Frederik Jorrit de Lange, Willem Petrus Merijn Emmanuël Hofland, Alessio Ferrara, Alessio Gargaro, Michele Brignole, Jan Gerrit van Dijk

**Affiliations:** Amsterdam UMC, Department of Clinical and Experimental Cardiology, Amsterdam Cardiovascular Sciences, University of Amsterdam, Heart Centre, Meibergdreef 9, 1105AZ, Amsterdam, The Netherlands; Amsterdam UMC, Department of Clinical and Experimental Cardiology, Amsterdam Cardiovascular Sciences, University of Amsterdam, Heart Centre, Meibergdreef 9, 1105AZ, Amsterdam, The Netherlands; BIOTRONIK Italia S.p.A., Clinical Research Unit, Via dell' Industria, 11, 20090 Milan, MI, Italy; BIOTRONIK Italia S.p.A., Clinical Research Unit, Via dell' Industria, 11, 20090 Milan, MI, Italy; Department of Cardiovascular, Neural and Metabolic Sciences, Faint & Fall Programme, IRCCS Istituto Auxologico Italiano, San Luca Hospital, Piazzale Brescia, 20, 20149 Milan, MI, Italy; Department of Neurology, Leiden University Medical Centre, Albinusdreef 2, Zuid Holland, 2333 ZA Leiden, The Netherlands

**Keywords:** Tilt table testing, Video, Finometer, Task force touch cardio monitor, Open access software

## Abstract

**Aims:**

We describe a novel, practical, and inexpensive method to add video recording during tilt table testing (TTT): Open-Access-Video-TTT.

**Methods and results:**

The Open-Access-Video-TTT set-up uses a personal computer (PC) to capture screen video data from a non-invasive-beat-to-beat (NIBTB) haemodynamic blood pressure (BP) device, combined with video recording of a patient, using Open Broadcaster Software (OBS®). The new Open-Access-Video-TTT set up was tested with both the Finometer (model Finapres Nova®, Medical Systems, the Netherlands) and the Task Force® Touch Cardio monitor (CNSystems, Austria). For this, the Finapres Nova® was enabled in ‘remote’ mode and Real Video Network Computing (RealVNC®) was installed on the PC/laptop. The Task Force® has a DisplayPort (DP) port, for which a DP/ high-definition multimedia interface (HDMI) cable and a video capture card is used to merge the signals to the PC/laptop. With this method the combined images are stored as a new video signal. TTT can be performed with any routine protocol.

**Conclusions:**

Open Access-Video-TTT worked well for both the Finapres NOVA® and the Task Force Monitor ®. This novel method can be used easily by all physicians who wish to add video recording during TTT who do not have access to an electroencephalogram machine.

**What’s new?**

- Novel implementation of video monitoring for tilt table testing.
- Implementation requires a personal computer, a video camera, and open access software.
- All physicians can now add video to tilt table testing.

## Introduction

Since the introduction of tilt table testing (TTT) in 1986,^1^ various TTT protocols have been reported with variation in the duration of the resting and tilted phases, tilt angle, type of support, and pharmacological provocation. In experienced hands, TTT is a valuable asset that improves syncope care through evoking witnessed events with recognition of complaints by the patient.^2,3^ Recent guidelines on syncope and on TTT recommended adding video recording to TTT, integrating video with non-invasive beat-to-beat (NIBTB) monitoring.^3,4^ Video recording during TTT allows an objective and repeatable review of clinical signs in relation to hemodynamic patterns, which increases the reliability of clinical observation of induced events. For example, in reflex syncope video recording of the actual moment of syncope helped distinguish between vasodepressor and cardioinhibitory mechanisms.^5^ Video recording also helped to reveal new pathophysiological insights in reflex syncope, such as that myoclonic jerks in syncope probably have a cortical origin.^6^ Video recording during TTT has also proved to aid diagnostic and therapeutic decision,^7,8^ when asystole starts after patients had already lost consciousness, asystole cannot be the primary cause of syncope, casting doubt of utility of pacing.^9,10^ Witnessed documentation of an attack, either with video recording during TTT, or with home video recording, is the gold standard to diagnose psychogenic pseudosyncope (PPS).^7,8,11,12^ Video recording of PPS during TTT ensures that apparent transient loss of consciousness occurs while blood pressure (BP) and heart rate (HR) are not low^7^ and thereby ensures the diagnosis. Video recording during TTT has helped confirm the combined presence of vasovagal syncope (VVS) and PPS, helping to distinguish VVS from PPS.^8^ A final advantage of video recording during TTT is that it allows physicians to review the semiology of tilt-evoked syncope at will, without having to be present during the entire TTT. See also *Table 1* for potential differences of TTT with video recording vs. without video recording.

**Table 1:** Potential difference of TTT *with* video vs. TTT *without* video

	TTT with video	TTT without video
Repeatable and objective review of clinical signs/T-LOC	Very easy	Impossible
Increase reliability of clinical observation	Very easy	Impossible
Timing of T-LOC with respect to haemodynamic parameters	Almost guaranteed	Very difficult
Diagnosing combined PPS and VVS	Very easy	Very difficult^a^
Establish diagnosis without physician attending TTT	Easy	Very difficult^a^
Assessing semiology of T-LOC	Possible	Difficult^a^
Biofeedback/professional education	Patients’ motions can be included	Patients’ motions cannot be included

Only possible with the presence of expert technician and only once.

Obtaining these advantages of video recording during TTT requires synchronization of the video signal with physiological signals such as HR and BP, acquired from an NIBTB monitor. Electroencephalogram (EEG) machines have been used for this purpose, as they offer synchronized video as standard, bringing combined haemodynamic and video-EEG monitoring within easy reach of neurologists. Unfortunately, the use of EEG machines is often impractical for other specialties.

We here describe a novel, practical and moreover not expensive method for video recording during TTT requiring a personal computer (PC), a video camera and open-source software that we call *Open-Access-Video-TTT.* We applied the method to 50 TTT to test for technical problems and to see whether standard operation procedures (SOPs) needed to be adjusted.

## Methods

The novel approach consists of capturing screen video data of the NIBTB device, combining this with a patient video, and storing the combined images as a new video signal. The new Open-Access-Video-TTT was installed and BP was recorded continuously, either with a Finometer (model Finapres Nova®, Medical Systems, the Netherlands) or with a Task Force® Touch Cardio monitor (CNSystems, Austria). TTT was performed according to the routine protocol available in either institute. Both types of NIBTB devices were set to depict the main hemodynamic parameters, i.e. BP and its determinants HR, stroke volume and total peripheral resistance, along with a one- or two-lead electrocardiogram (ECG).

**Figure 1:**
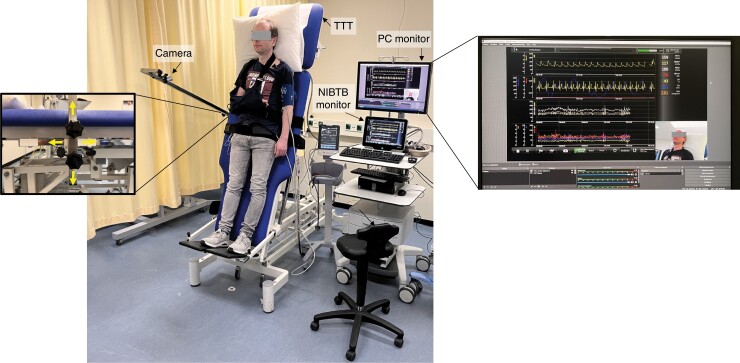
Overview in reality of the set up open-access-video-TTT with finapres NOVA® as example (recorded in Amsterdam, WH on the picture and gave informed consent). Arrows pointing at different instruments used. Detailed view on the right side shows the picture-in picture of the patient’s head and shoulders and its vital parameters. Detailed view on the left side shows the possibilities to adjust the camera position, mounted on the tilt table, to the height of the patient. TT, tilt table.

A video camera was either mounted to the tilt table, so it moved up and down with patients while remaining aimed at patients’ head and shoulders or was ceiling-mounted, providing an overview of the entire tilt table. We have no preference for either camera position; one choice ensures continuous detailed views of the face but ignores limb movements; the other allows a whole body overview (*Figure 1* shows an example of the camera position mounted on the tilt table).

A PC or laptop was used to capture screen data from both the NIBTB device and the video camera recording patient activity (*Figure 1*).

We installed Open Broadcaster Software (OBS®; https://obsproject.com/) on the PC/Laptop to add a live picture-in picture of the patient video to the captured screen data of the NIBTB device. OBS® allows to save and replay the recorded signal as picture in picture on the PC/laptop.

The main difference between the Finapres NOVA® and the Task Force® monitor is the ‘remote function’, not available on the Task Force Monitor.

### Finapres NOVA® device

Besides Open Broadcaster Software, we installed Real Video Network Computing (RealVNC®, available for download on: https://www.realvnc.com/en/) and Open Broadcaster Software (OBS® available for download on: https://obsproject.com/) on the PC/laptop.

To do so, the Finapres NOVA ® device was first connected to the PC/Laptop by a regular network cable. The camera video output was transferred to the PC/Laptop using a USB 3.0 cable.

We then enabled the ‘remote’ setting on the Finapres NOVA® device, after which video computing network (VNC) could capture the Finapres NOVA® screen signal and show it on the PC (*Table 2*).

**Table 2:** Hardware and software needed for open-access-video-TTT

**Finapres NOVA® device**
• Finapres Medical Systems Finometer
• PC/laptop with Windows, Linux or Mac
• USB Camera (1080p P)
• LAN/USB 3.0 cables as needed
• Real Video Network Computing. RealVNC®, available to download on: https://www.realvnc.com/en/
• Open Broadcaster Software. OBS Studio, available to download on https://obsproject.com/
**Task Force® device**
• CNSystem® Task Force Monitor
• Laptop with Windows, Linux or Mac
• CCTV dual mode camera (Visible and InfraRed light spectrum), with 2MPx (1080P)
• HDMI/DP cables as needed
• 4 Channel Digital A/V USB3.0 HDMI Video Capture Card
• AV composite RCA to HDMI adapter
• Open Broadcaster Software. OBS Studio, available to download on https://obsproject.com/

**Figure 2:**
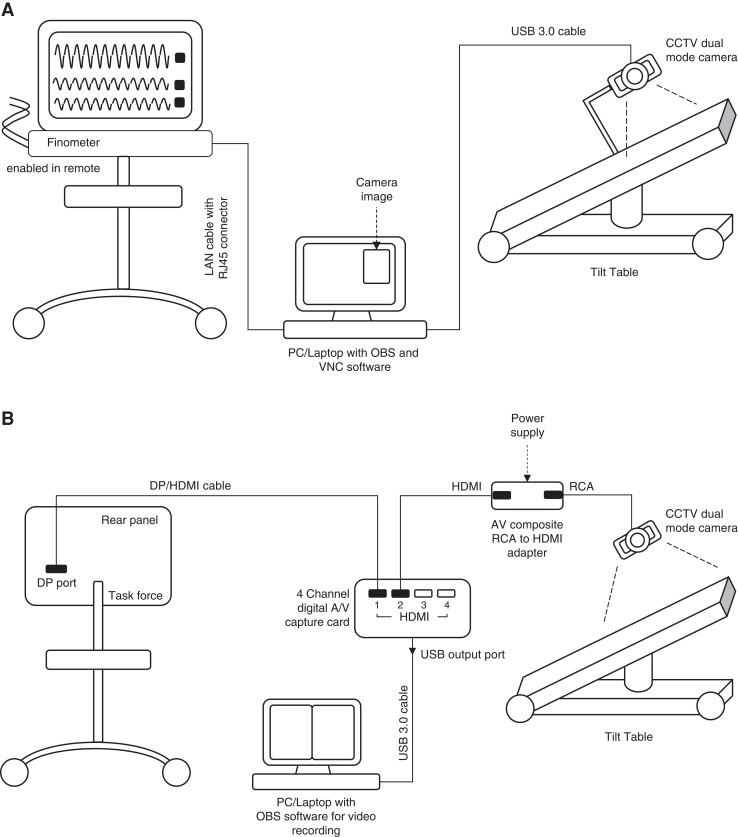
Schematic drawing of the set up for open-access-video-TTT with a finapres NOVA® device (*A*) and task force® monitor device (*B*). CCTV, close-circuit television; LAN, local area network; TT, tilt table; USB, universal series bus connector.

Once complete, OBS® was used to add a live picture-in picture of the patient video to the PC screen (*Figure 2A*).

### Task force® device

The Task Force® device does not have ‘remote’ function but has a DP port. Hence, a DisplayPort (DP)/high-definition multimedia interface (HDMI) cable is used to connect the DP port at the rear panel of the Task Force® monitor to the first HDMI channel of the video capture card.

Then, the video camera is connected to the second HDMI port of the video capture card through an adapter [audio video (AV) composite Radio Corporation of America (RCA) to HDMI adapter] with an RCA connector input (for video signal) and a HDMI output. The video capture card merges the two video signals, i.e. from the Task Force® monitor and the camera that are displayed on the PC monitor.

Finally, the output of the video capture card is connected to the PC/laptop through a USB 3.0 cable (*Table 2*).

OBS® was then used on the PC/Laptop as before (*Figure 2B*).

## Results

### Applicability in daily practice

After installing the open-access software and changing the SOP, the novel method Open-Access-Video-TTT proved easy to operate in daily practice, both for technicians and physicians (see *Figure 1* for a detailed overview of the complete setup of the Open-Access-Video-TTT). The first 50 TTT were carried out without technical problems: apart from connecting the device the TTT operating procedures were not affected.

### Open-access-video-TTT changes routine practice: aspect of reviewing the TTT

We reviewed the video as often as needed, meaning we quickly reviewed tests in which nothing happened, and spent more time reviewing tests in which something of interest happened. We decided to have physicians review the tests together with technicians, which adds experience and helps improve practice.

We have found that reviewing the video can change the report or conclusion of the TTT. For example, complaint recognition may alter the clinical diagnosis. A technician recently stated that a patient had reported ‘no complaint recognition’ concerning a classical mixed vasodepressive and cardioinhibitory syncope during TTT. However, the technician and physician reviewed the video together and heard the patient mention ‘heart cramp’ just before syncope; the patient also mentioned on the video that she was very tired and weak afterwards. The physician discussed these specific items during the next consultation, whereupon the patient mentioned that the event during TTT was infact the same as in daily life, but developed faster during TTT. Recognition on TTT is a very important clinical aspect that helps confirm the diagnosis and improves the value of TTT.^2,3^ This degree of subtlety would have been impossible without reviewing the video. We now routinely record questions and answers about complaint recognition.

An instruction video of the novel method Open-Access-Video-TTT is also available as a Supplementary material online, *supplementary file* in this journal.

## Discussion

We described a novel, easy and inexpensive method to integrate synchronized hemodynamic and video recording during TTT: *Open Access-Video-TTT* for either a Finapres NOVA® or a Task Force Monitor ®. The method can be used by all physicians who wish to add video recording during TTT who do not have access to an EEG machine.

### Video on TTT provides biofeedback for both physicians and clinical researchers

Video recording during TTT in suspected syncope increases the reliability of clinical observation of induced events for physicians, who can review the course of events as often as needed.

Without recording much information will be lost or cannot be recalled. Understanding what happened during TTT is of paramount importance for clinical reasoning.^9,10^ Reviewing haemodynamic parameters of a syncopal event in relation to the clinical events, such as patients’ movements, will provide much insight in the pathophysiology of the event. In turn, this will train physicians to become more alert of such events, which will improve history taking. For example, experience with video taught that patients making sounds or exhibiting roving eye movements during syncope indicates severe hypoperfusion and probably asystole.^6^

Publication of the SPAIN,^13^ BIOSYNC-trial^14^ results encouraged clinical researchers to perform a in depth analysis with respect to the moment of loss of consciousness during a syncopal event on TTT.^15^ Of particular importance is whether asystole starts before or after the onset of loss of consciousness.^9^ Having all vital tracings synchronous with a video has already yielded more insight in cardioinhibition during reflex syncope, with implications for pacing. Open-Access-Video-TTT will help BioSync CLS trial investigators and others to better understand the role of CLS in pacing for the older patient with recurrent reflex syncope.

### Management consequences for a syncope unit

Current syncope guidelines do not require a physician to attend the tilt table test^4,16^ for safety considerations. While true, this ignores the didactic elements of being present. Video recording fills this gap, allowing professionals to study detailed events with very little effort. Adding video to the TTT also makes it easier for nurses or technicians to perform TTT without the presence of a physician. Performing biofeedback using video recording can also be applied in more complex cases and can be performed by trained nurses or technicians. Video recording therefore will probably have an impact on the costs of performing TTT in the management of syncope.^17,18^

### Ethical and legal matters

We suggest that those wishing to add video to TTT should check local regulations regarding making a video record of a patient. Note that video during TTT should be considered as an integral part of routine standard patient care, just as in video-EEG, for which different rules may hold than for use of video material in medical education or for purely scientific purposes. In the latter case informed consent by the patient may well be necessary, depending on local regulations. We have added information to the general TTT information that a video is part of standard routine medical care. No patient has yet refused video recording for healthcare purposes.

### Open-access-video-on-TTT vs. video-EEG monitoring during TTT

Video recording during TTT was until now mainly performed by neurologists using an EEG machine. In the LUMC (Leiden University Medical Centre, The Netherlands) video recording during TTT has been performed since about 2000, resulting in thousands of tests. EEG machines can store and replay synchronous continuous data consisting of the EEG, one or more ECG leads, any additional analogue or digital signal, and a video signal. Hence, adding an EEG machine to TTT offers more recording and reviewing opportunities than video-Open-Access-TTT, but at the obvious expense of acquiring and operating an EEG machine.

We emphasize that a complete video-EEG registration is considered superior to video only when it comes to recognize PPS^2,3^ or psychogenic non-epileptic seizures (PNES). When PNES is probable *a priori*, a test with video-EEG should preferentially be ordered. For PPS, the EEG may probably be supplanted by near-infrared spectrometry or Transcranial Doppler. If such events occur unexpectedly, the video may be shown to those with relevant expertise.

The advantage of an EEG during syncope does not lie in detecting the earliest signs of cerebral hypoperfusion, as the EEG only responds late during developing hypoperfusion. However, the EEG then does so quickly and impressively, with a loss of normal activity and the occurrence of coarse abnormal activity (delta waves or electrical silence). The EEG is never normal during syncopal loss of consciousness, because the EEG mainly reflects cortical activity, the cortex is very susceptible to hypoperfusion, and because loss of consciousness in syncope is due to an overall profound loss of cortical function.^6^ Recording EEG signals during TTT indicates when cortical function becomes severely impaired, teaching physicians which signs point at Loss of Consciousness, when to tilt back, and helps distinguish between syncope and PPS, as the EEG during PPS typically shows a completely normal ‘awake eyes closed’ pattern. The EEG is particularly useful in cases of syncope morphing into PPS or vice versa.^8^

An advantage of video-EEG reviewing is that EEG machines enable the user to change the time base of the recording, to zoom in on events lasting a few seconds, or to zoom out to obtain an overview of an entire syncopal period. In addition, most signals can be filtered digitally, which helps to reduce artefacts and allows scrutiny of specific frequency signals, such as EEG slowing.

We believe that adding the novel video method to TTT will increase appreciation of the great utility of video recording to better understand paroxysmal events. In epilepsy, it is at present unthinkable to perform an EEG without video; the same situation may develop for TTT. Seeing patients with T-LOC in all its forms requires not only a structured approach to assess what happened during an event, but benefits from a multidisciplinary approach, with access to appropriate diagnostics and professionals. We therefore we hope that all syncope clinics applying this novel method will benefit from it.

## Supplementary Material

euac193_Supplementary_Data

## Data Availability

The data underlying this article are available in the article and in its online supplementary material.
